# Proteomic Profiling of Density-Gradient Fractions Reveals Candidate Cell Surface Markers in Subpopulations of Starfish *Asterias rubens* Coelomocytes and Coelomic Epithelial Cells

**DOI:** 10.3390/ijms27146292

**Published:** 2026-07-15

**Authors:** Sergey V. Shabelnikov, Natalia S. Sharlaimova, Dan E. Bobkov, Alexey G. Mittenberg, Olga Petukhova

**Affiliations:** Institute of Cytology of Russian Academy of Sciences, 194064 St. Petersburg, Russia; sergey.v.shabelnikov@gmail.com (S.V.S.); nashar@yandex.ru (N.S.S.); bobkovde@yandex.ru (D.E.B.); mittenberg@incras.ru (A.G.M.)

**Keywords:** echinoderm, *Asterias rubens*, coelomocytes replenishment, coelomic epithelium, cell fractionation, membrane proteome, LC-MALDI, Percoll density gradient

## Abstract

Cell surface markers are essential for lineage tracing and for understanding cellular mechanisms of regeneration, yet they remain largely unavailable for echinoderms. Using the starfish *Asterias rubens* as a model for physiological coelomocyte renewal, we combined Percoll density-gradient fractionation with label-free LC-MALDI proteomics to identify candidate surface proteins that distinguish subpopulations of coelomocytes and coelomic epithelium (CE) cells. However, LC-MALDI is prone to signal variability, sample depletion and nonlinear response. To address the quantitative limitations of LC-MALDI, we implemented a chi-square test with an empirically derived probability density function, which we validated on control mixtures, achieving high precision. Four fractions enriched in distinct cell types were analyzed: coelomocyte fractions 1 (roundish cells) and 4 (large agranulocytes that form cell nets) and CE fractions 1 (type 2 small CE cells) and 4 (ciliated cells), together with a weakly attached CE subpopulation enriched in small putative progenitor cells. LC-MALDI identified 714 protein groups, of which 160 showed significant differential abundance across fractions. Functional enrichment revealed actin cytoskeleton and integrin signaling in coelomocytes, and catenin-related terms in CE-W. Among the 101 predicted membrane proteins, 31 candidate cell surface markers were selected. These include integrins α8β1 and α9β1, three-finger proteins, scavenger receptors, and low-density lipoprotein receptor-related proteins, as well as metallopeptidases. This work provides the first proteome-scale set of candidate surface markers for starfish cell subpopulations, enabling future lineage-tracing studies to resolve the origin of coelomocytes from the coelomic epithelium.

## 1. Introduction

Echinoderms, a phylogenetically ancient group of non-chordate deuterostomes, possess remarkable regenerative capacities, being able to restore lost body parts and internal organs. This regenerative process relies on cellular mechanisms that ensure tissue homeostasis, wound healing, and replacement of damaged cells. Key questions in echinoderm biology include identifying the specific cell types responsible for regeneration, determining whether they are dedicated stem cells or dedifferentiated somatic cells, and understanding their origin [1]. Most experimental data support dedifferentiation or transdifferentiation of somatic cells, and epithelial morphogenesis, as the main regenerative mechanisms. In contrast, the existence and role of adult stem cells remain controversial [1,2,3].

Our research focuses on physiological regeneration—the continuous replacement of cells that maintains normal tissue composition—in the coelomic cavity of the starfish *Asterias rubens*. The coelomic cavity is populated by free-moving cells known as coelomocytes. The replenishment of coelomocytes in *A. rubens* is particularly convenient for studying physiological regeneration, as the coelomic fluid can be experimentally drained, allowing the dynamics of cell pool recovery to be tracked in vivo. It has long been hypothesized that mature coelomocytes originate from the coelomic epithelium (CE) that lines the cavity [4]. We have previously suggested that coelomocytes are recruited to the coelomic fluid through two routes: (i) detachment of mature coelomocytes adhering to the CE surface (marginal coelomocytes) and (ii) migration of small, undifferentiated cells from the CE [5]. Marginal coelomocytes were identified as cells washed out of the coelom after the coelomic fluid had been drained as completely as possible. In intact animals, this cell population contains a significant proportion of large agranulocytes. Two types of small epithelial cells with a high nuclear-to-cytoplasmic ratio have been identified in the CE: type 1 small epithelial cells (SECs-1), whose nuclei stain discretely with DAPI, and type 2 small epithelial cells (SECs-2), with densely stained nuclei. Of these two cell types, only SECs-1 are contained in the coelomic fluid, and a subpopulation of weakly attached CE cells (CE-W) enriched in SECs-1 can be isolated during CE preparation without further fractionation [5,6]. Based on morphology and the presence of dividing cells, SECs-1 have been proposed as coelomocyte precursors [7]. According to morphometric characteristics, these cells correspond to lymphocyte-like or progenitor cells from other authors [8,9,10]. Nevertheless, the origin of SECs-1 on the CE surface remains unclear: do they derive from resident stem cells, or do they arise from dedifferentiation and migration of specialized epithelial cells? A major obstacle to answering this question is the lack of lineage-specific cell surface markers that would enable tracking of SECs-1 differentiation into mature coelomocytes.

To address this gap, we designed a strategy combining density-gradient fractionation, morphological characterization, membrane fraction isolation and label-free proteomics. Specifically, we used a six-step Percoll density gradient to obtain subpopulations of coelomocytes and CE cells enriched in particular morphotypes, including fractions 1 and 4 of coelomocytes (Coe1, Coe4), fractions 1 and 4 of CE (CE1, CE4), and the CE-W subpopulation enriched in SECs-1. For protein identification and quantification, we employed LC-MALDI MS/MS, which was the method of choice given its availability in our laboratory and its compatibility with the analysis of complex peptide mixtures. However, it is widely acknowledged that LC-MALDI is not inherently suited for quantitative proteomics. MALDI ionization is prone to signal variability due to uneven co-crystallization of the matrix and analyte [11]. Nevertheless, several studies have demonstrated that with appropriate workflow optimization LC-MALDI can yield quantitative results comparable to ESI-based approaches [12,13]. Given these inherent limitations of LC-MALDI for label-free quantification, we used the sum of peptide-spectrum matches (PSMs) for a given protein as a proxy for protein abundance; this metric, in our experience, is less susceptible to fluctuations than absolute peak intensities. Furthermore, to accommodate the limited number of replicates we implemented and validated statistical framework based on the chi-square statistics with an empirically derived probability density function (ePDF). This approach allowed us to confidently identify differentially abundant proteins across cell fractions.

Given the central hypothesis that the CE, particularly the SEC-1 cells in the CE-W subpopulation, gives rise to circulating coelomocytes, we designed this study to address three specific aims. First, to implement and validate a label-free quantification method for spectral count data obtained with LC-MALDI. Second, to characterize the proteomes of coelomocyte and CE fractions enriched in distinct morphotypes, including the putative precursor-enriched CE-W population. Third, to nominate candidate cell surface markers that can distinguish coelomocytes, CE cells, and the presumptive precursor-enriched CE-W subpopulation. The results provide a first-generation molecular toolkit for lineage tracing in *A. rubens* and open the way to understanding the cellular basis of physiological regeneration in echinoderms.

## 2. Results

### 2.1. Percoll Density-Gradient Fractionation Enriches Distinct Morphotypes with Differential Functional Behavior

Following fractionation of coelomocyte and coelomic epithelium (CE) cell suspensions on a six-step Percoll density gradient, five cell fractions were obtained from each. A detailed description of the composition of each fraction was provided in a previous study [14]. In this analysis, cell fractions enriched in specific morphotypes were selected for morphological and proteomic assessment. We focused on fractions 1 and 4 of coelomocytes (Coe1 and Coe4), fractions 1 and 4 of CE (CE1 and CE4), and a subpopulation of CE-W cells enriched in poorly differentiated cells isolated without further enrichment procedures.

**Figure 1:**
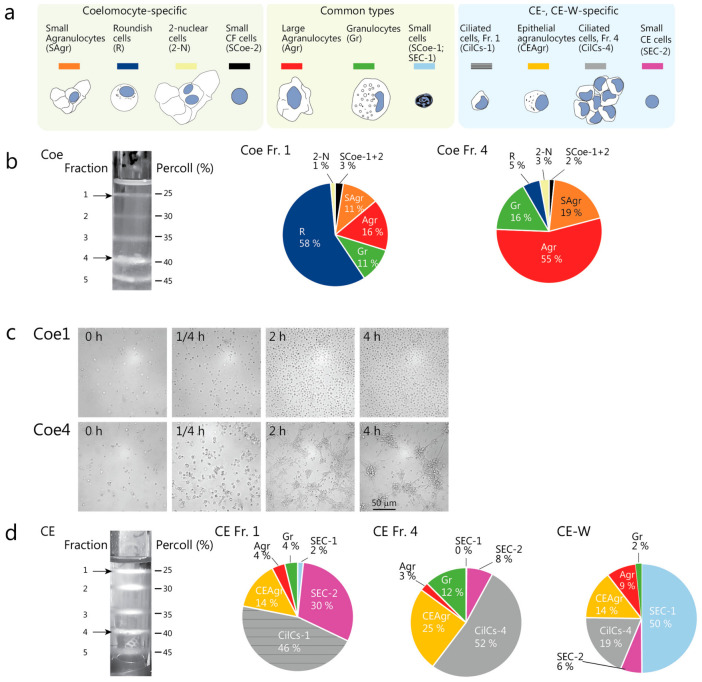
Cellular composition of Percoll density-gradient fractions. (**a**) Cell morphotypes identified in the coelomocyte (Coe) and coelomic epithelium (CE) fractions, and in weakly attached CE cells (CE-W). (**b**) Relative abundance of cell types in coelomocyte fractions 1 (Coe1) and 4 (Coe4). Fractions SCoe1 and SCoe4 were pooled due to their low abundance. (**c**) Functional assay: Coe1 and Coe4 cells incubated in seawater for 4 h. Note the aggregation and nets formation in Coe4, whereas Coe1 remains as single cells. (**d**) Relative abundance of cell types in CE fractions 1, 4 and CE-W. Left panels in (**b**,**d**) illustrate representative Percoll separation.

The various cell morphotypes, including those specific to coelomocytes and CE, as well as shared cell types, are presented in Figure 1a. A comprehensive description of cell morphology and compositional statistics can be found in Tables S1 and S2. The cellular composition of the selected Percoll fractions is depicted in Figure 1b,d. The dominant cell type in Coe1 consists of roundish cells (R) with fine-grained regions in the cytoplasm and densely stained nuclei (d_n_ 4.3 ± 0.1, d_c_ 8.2 ± 0.2), making up 58% of the population. In contrast, Coe4 is enriched in large petaloid agranulocytes (55%), (Agr) with densely stained round nuclei (d_n_ 4.18 ± 0.7, d_c_ 11.7 ± 0.7 in CF; d_c_: 9.5 ± 0.33 in CE and CE-W). This cell type was identified in both CE and coelomocyte preparations. Functional tests conducted on coelomocyte fractions during incubation in seawater demonstrated that Coe4 is capable of in vitro cell net formation, unlike Coe1 (Figure 1c, Video S). The dominant cell types in CE1 and CE4 were small agranulocytes with irregular nuclei (d_n_ 3 ± 0.5, d_c_ 4.8 ± 0.17), constituting 46% and 52%, respectively (Figure 1d). These cells have previously been identified as CE ciliated cells [14]. The key difference is that ciliated cells of fraction 4 tend to form cell aggregates, unlike those of fraction 1. Additionally, CE1 was enriched with small epithelial cells with a high nuclear-to-cytoplasmic ratio featuring densely stained nuclei, known as SEC-2 (30%, d_n_ 2.8 ± 0.2 d_c_ 3.3 ± 0.2). In the subpopulation of CE-W cells, the dominant cell type was small epithelial cells with a high nuclear-to-cytoplasmic ratio and discretely DAPI-stained nuclei and invisible cytoplasm (SECs-1, 50%). Round (4.06 ± 0.2) and oval (4 × 6.06 ± 0.5) nuclei were found (Figure 1d).

### 2.2. A Chi-Square-Based Empirical Probability Density Function Enables Label-Free Quantitation in LC-MALDI Data

**Figure 2:**
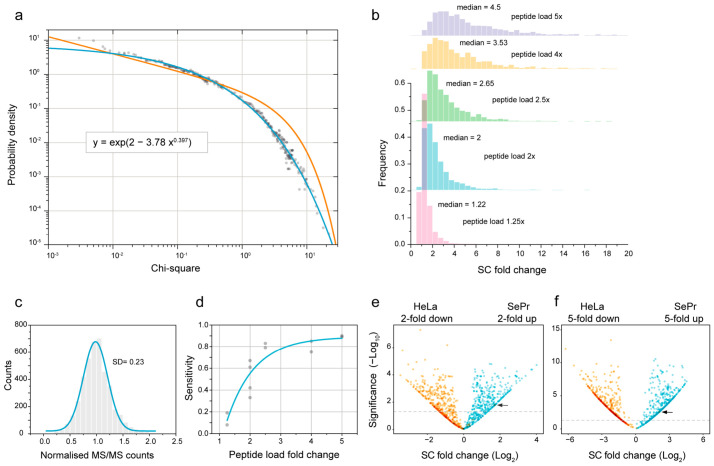
Validation of the chi-square-based empirical probability density function (ePDF) for label-free quantitation. (**a**) Comparison of the empirical chi-square distribution (blue) with the theoretical chi-square distribution (k = 1, orange) on a log–log scale. (**b**) Distributions of spectral counts fold-change at different true peptide load fold change. Note correlation of medians with peptide load. (**c**) Distribution of spectral counts normalized across replicates. (**d**) True positive rate at *p* < 0.05 as a function of true peptide load fold change. (**e**,**f**) Volcano plots showing differential protein abundance in HeLa (brown) and *S. proteamaculans* (SePr, blue) digest mixtures; dashed line indicates significance threshold (*p* = 0.05). Arrow marks false positive.

Here, we derived an empirical probability density function (ePDF) for the chi-squared statistics for use in LC-MALDI label-free proteomics. The approximation of the experimental data is given by the stretched exponential decay and shows deviation from the classical chi-squared distribution with one degree of freedom (Figure 2a). Critical chi-square values for our test are 2.47, 5.5 and 12.08 for *p*-values of 0.05, 0.01 and 0.001, respectively. We used averaging of spectral counts across replicates because the counts are distributed normally (Figure 2c). The true positive rate (TPR) was evaluated at the significance level of 0.05. It shows that our method possesses relatively low sensitivity, reaching 90% TPR only at more than a 4-fold difference in peptide load (Figure 2d). However, as seen from Figure 2e,f, the method is precise since only a few false positives were observed. From our observations, there is a nonlinear relationship between spectral count (SC) fold change and peptide load fold change for a particular protein. Nevertheless, the median values of SC fold change distributions correlate well with peptide load fold change (Figure 2b).

### 2.3. Proteomic Landscapes Reveal Divergent Functional Signatures and Candidate Surface Markers

**Figure 3:**
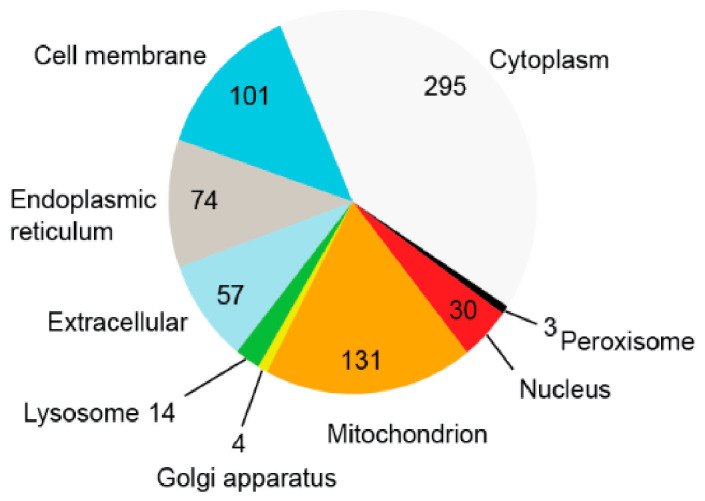
Predicted subcellular localization of the 714 identified proteins across all fractions.

**Figure 4:**
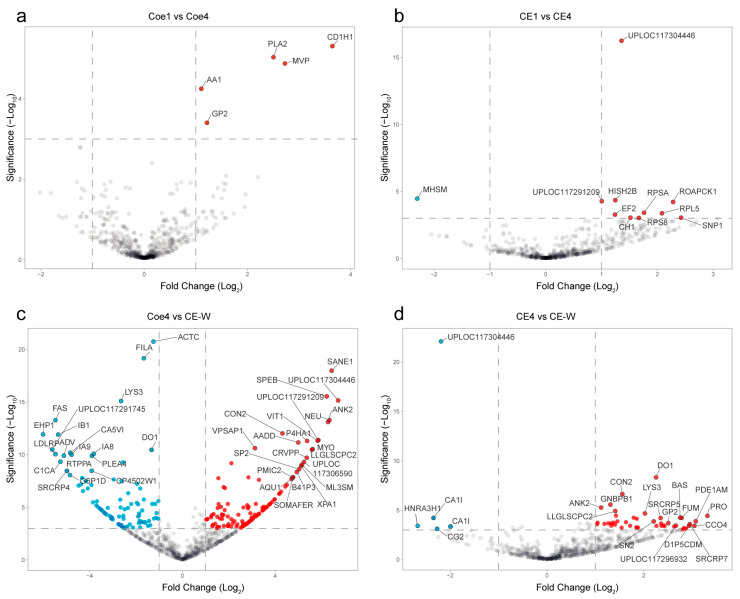
Comparative proteomic analysis of coelomocyte and coelomic epithelium fractions. Volcano plots displaying differentially abundant proteins between (**a**) Coe1 vs. Coe4, (**b**) CE1 vs. CE4, (**c**) Coe4 vs. CE-W, and (**d**) CE4 vs. CE-W. Significance was determined using the chi-square test with ePDF (*p* < 0.001). Proteins with SC fold change > 2 are highlighted. Coe, coelomocytes; CE, coelomic epithelium; CE-W, subpopulation of weakly attached CE cells. The horizontal dash lines—2-fold change, vertical dash lines—0.001 significance.

LC-MALDI identified 714 protein groups across all selected fractions (Table S3). These proteins were categorized into nine subcellular locations (Figure 3), with 101 proteins assigned to the cell membrane category. A comparison of coelomocyte fractions Coe1 and Coe4 revealed only minor differences (Figure 4a). The notable difference was the significant upregulation of alpha-actinin (AA) and plastin (PLA2) in Coe4, suggesting variations in actin cytoskeleton regulation as well as upregulation of dynein, a motor protein associated with the tubulin cytoskeleton. The CE fractions CE1 and CE4 also displayed minor differences (Figure 4b). Only myosin heavy chain (MHSM) was upregulated in CE1, whereas CE4 included 10 upregulated proteins, the most abundant of which was an uncharacterized protein similar to a microtubule-associated protein (UPLOC117304446 (MACF1)).

A comparison of CE-W proteins was performed only with the Coe4 and CE4 fractions, as the protein pools of Coe1 and CE1 differ only slightly from those of Coe4 and CE4, respectively (Figure 4a,b). The comparison between Coe4 and CE-W revealed significant differences in their protein sets (Figure 4c), while the protein pools of CE4 and CE-W exhibited differences to a lesser extent (Figure 4d). Consistent with the comparison between CE1 and CE4, CE4 showed a higher abundance of a microtubule-associated protein relative to CE-W.

**Figure 5:**
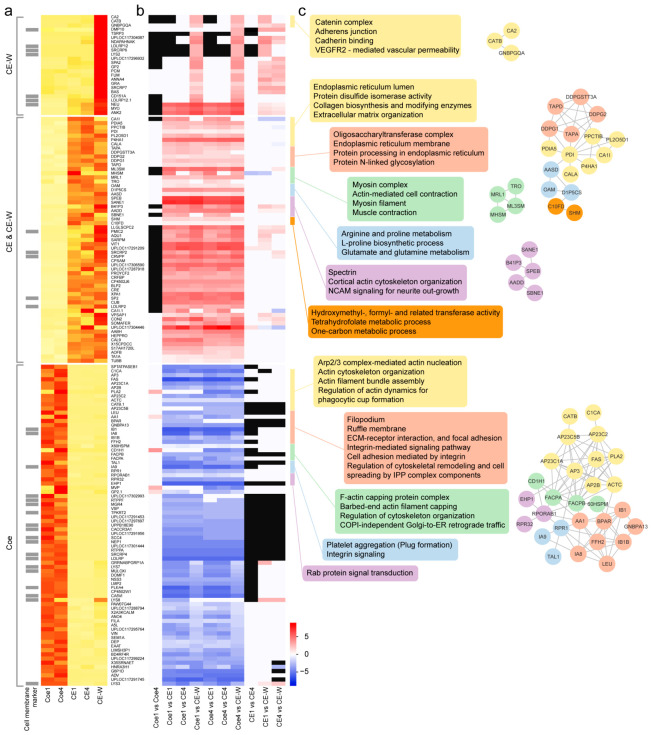
Identification of candidate cell surface markers and functional enrichment. (**a**) Heatmap of 160 differentially abundant proteins (*p* < 0.001) across fractions. Rows are z-scored; grey boxes indicate suggested cell surface markers. (**b**) Heatmap of log2 spectral count ratios between fractions. Only significant differences (*p* < 0.001) are colored; white indicates non-significant, black indicates protein not detected. (**c**) STRING enrichment networks for proteins upregulated in coelomocyte (Coe), coelomic epithelium (CE), and weakly attached CE cell (CE-W) subpopulations. Node colors represent functional categories.

For further analysis, we selected proteins that exhibited differential abundances in individual cell fractions, resulting in a list of 160 proteins. Next, we performed enrichment analysis using STRING to identify biologically relevant processes that would provide meaningful associations with the distinct cell fractions (Figure 5).

The CE fraction is defined by a conspicuous enrichment of endoplasmic reticulum (ER) and Golgi-resident folding and glycosylation machinery, together with secreted ECM components. The core signature includes collagen α-1 chains (CA1I) and components of its maturation pathway (P4HA1, PL2O5D1; three subunits of the oligosaccharyltransferase (OST) complex (DDPGSTT3A, DDPG2, DDPG1) and other proteins that further attest to a high flux of maturing collagens and cysteine-rich secretory proteins (PDI, PDIA5, PPCTIB). Several proteins exhibit a secretory phenotype (CUB, VIT1, CRFBP and a number of uncharacterized secreted factors), while metabolic enzymes (OAM, D1P5CS, CPSAM) indicate biosynthesis of proline and polyamines required for collagen production. Overall, CE cells function as a specialized secretory and structural scaffold, focused on fibrillar collagen production and ECM assembly.

The CE-W fraction balances adhesion, signaling and limited ECM interaction. Two catenins (CA2 and CATB) form a cadherin-associated module that links the plasma membrane to the actin cytoskeleton. Their co-expression with some recognizing and actin-associated proteins (ANK2, GP2, MYO and ANNA4) suggests dynamic actin remodeling, while CATB indicates its involvement in the Wnt signaling pathway.

The membrane proteome is rich in receptors that integrate adhesive and migratory cues: DMP10 or ADAM10, two LDLRP12, NEU, CD151A, BAS and SRCRP superfamily proteins. The presence of the GPI-anchored Ly6/uPAR family member LYS2 further diversifies surface recognition. The heterotrimeric G protein subunit G-q GNBPGQA indicates G-protein-coupled receptor signaling capacity. CE-W cells also harbor some of the ECM-synthetic machinery characteristic of CE cells. Thus, CE-W cells appear primed for adhesion and migration as well as local signal integration, with potential for ECM production.

The Coe fraction exhibits a proteome dominated by regulators of actin dynamics, integrin-based adhesion and directional migration, hallmarks of a motile, migratory phenotype. Actin itself (ACTC) was accompanied by the entire Arp2/3 complex (AP23C1A, AP2B, AP23C5B) and actin-related proteins (AP2B and AP3), nucleation-promoting factors (FAS) and filament-severing/-capping proteins (GP2, DEP, ADV, FACPA and FACPB). Additionally, this fraction is enriched in crosslinkers and stabilizers (FILA, PLA2, AA1, C1CA), focal adhesion proteins (IB1, IB1B, IA8, IA9, TAL1, VIN, FFH2, LEU, BPAR) and small GTPase signaling proteins for adhesion turnover and migration (the ras-related proteins RPR1 and RPORAB1, G-family protein GNBPA13 and cell adhesion and motility protein VSP). Notably, the Coe population expresses three proteins with prominent roles in directional migration and inflammatory lipid signaling (PLEA4, SEM1A, A5L).

Additional signaling and remodeling molecules that emphasize the mobile, invasive nature of Coe were also identified (RTPPF-like and RTPPA-like, known to regulate cadherin and integrin adhesion; CATB and NEP1, involved in ECM degradation; the ROS-generating system (MULOXI and DOMF1); receptor tyrosine kinase (TPKRT2); and G-protein-coupled receptor (MGR4)). Collectively, the Coe proteome defines a highly motile, adhesion-competent cell type with the machinery for both chemotactic signaling and responses to soluble ligands.

**Figure 6:**
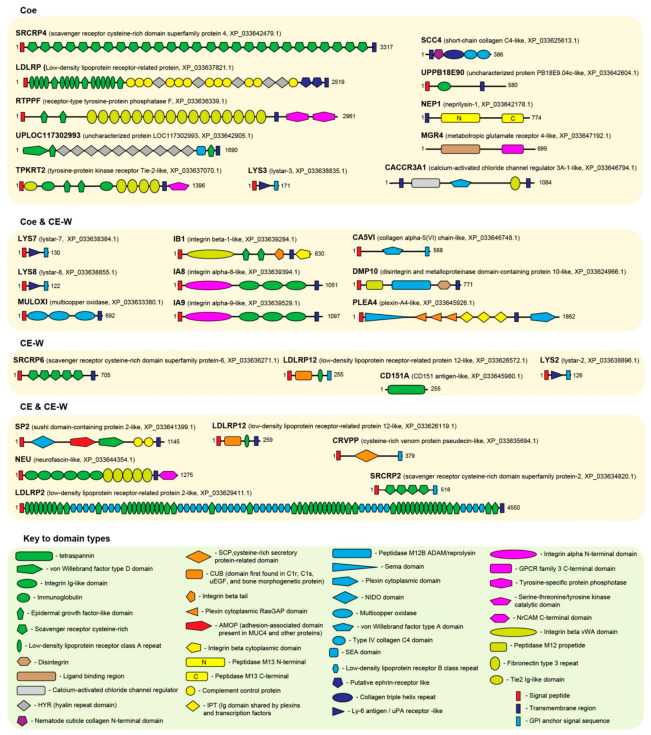
Domain organization of proposed cell surface markers. Coe—coelomocytes, CE—coelomic epithelium, CE-W—weakly attached CE cells. The piezo-type mechanosensitive ion channel component 2-like protein (PMIC2, XP_033625758.1) is not shown in the diagram.

Finally, we have selected 31 potential cell surface marker candidates (Figure 5a) that met the following criteria: (1) predicted cell membrane localization, (2) significant shifts in protein abundance, and (3) absence in one or more analyzed cell fractions. The domain organization of these proteins is presented in Figure 6. Among the selected proteins, 15 are single-pass receptors without a cytoplasmic domain, including three metallopeptidases: disintegrin and metalloproteinase domain-containing protein 10-like (DMP10), neprilysin-1 (NEP1), and calcium-activated chloride channel regulator 3A-1-like (CACCR3A1). Other proteins are putatively classified as pattern-recognition receptors and opsonins. Five putative markers are single-pass proteins with a cytoplasmic signaling domain, comprising the adhesion receptors integrin alpha-8-like (IA8), integrin alpha-9-like (IA9), integrin beta-1-like (IB1), integrin beta-1-B-like (IB1B), neurofascin (NEU), plexin-A4 (PLEA4), receptor-type tyrosine-protein phosphatase F-like (RTPPF), and the putative angiopoietin receptor (tyrosine-protein kinase receptor Tie-2-like, TPKRT2). The remaining surface markers include seven GPI-anchored proteins, among which three-finger proteins are of special interest, and three multipass proteins, including a G-protein-coupled glutamate receptor (MGR4), CD151 antigen-like (CD151A) and piezo-type mechanosensitive ion channel component 2-like (PMIC2).

## 3. Discussion

In this study, we combined density-gradient fractionation, morphological characterization, membrane protein isolation, and label-free LC-MALDI proteomics to identify candidate cell surface markers for distinct subpopulations of coelomocytes and coelomic epithelium (CE) cells in the starfish *A. rubens*. Previous proteomic studies of echinoderms include several conducted on Asteroidea. A functional review of identified proteins in *Marthasterias glacialis* coelomocytes highlights the diverse roles these cells play in echinoderm biology. These proteins provide preliminary evidence for several molecular pathways, including cytoskeletal regulation and cell adhesion, signaling, cell regulation and proliferation, and regeneration [15]. Proteomic characterization of two distinct stages of neuronal regeneration in the starfish *Marthasterias glacialis* after tentacle tip amputation revealed differential protein abundance in the soluble and membrane-enriched fractions [16]. The authors emphasize the involvement of various molecular mechanisms in this process: proteolysis as a post-translational modification, the dynamics of the actin and tubulin cytoskeleton, vesicular transport, and the ubiquitin–proteasome system in the regulation of protein levels. The proteome of mucous and adhesive secretions from the starfish *A. rubens* identified proteins involved in defense and adhesion [17]. Proteomic analysis of CE from total water-soluble extracts shows that CE cells are actively involved in protein synthesis and processing, as well as membrane transport processes such as phagocytosis and mass secretion, intercellular adhesion, ciliary motility, and the ubiquitin–proteasome system [18]. Unlike the above-mentioned works, we analyzed the proteomes of membrane components from individual cell fractions, each enriched in distinct cell types. Our approach revealed 714 protein groups, 160 of which showed significantly differential abundance across fractions, leading to the nomination of 31 putative surface markers. The obtained results provide a first-generation molecular toolkit for tracking cell differentiation in *A. rubens* and offer insights into the cellular mechanisms of physiological regeneration.

The study is based on proteomics and bioinformatics data, so the identified differentially expressed proteins are considered only as candidates for markers of individual cell types. The potential use of the most promising candidates as marker proteins will be confirmed using PCR, FISH analysis, and immunofluorescence analysis.

### 3.1. Quantitative Performance of the ePDF-Adjusted Chi-Square Test

Protein quantification with LC-MALDI is challenging because conventional label-free quantification workflows are predominantly developed for electrospray ionization platforms. Here we adapted a chi-square statistical method with an empirically derived probability density function (ePDF) to control false positives in LC-MALDI data. This adjustment reduced the risk of overestimating differential expression—a common pitfall when applying standard statistical tests to spectral count data. Our validation using mixed digests of HeLa and the Gram-negative bacterium *S. proteamaculans* demonstrated that the method achieves high precision (very few false positives) but only moderate sensitivity: a >4-fold change in peptide load was required to reach 90% true positive rate. This trade-off is acceptable for discovery-phase proteomics, particularly when the goal is to identify robust, large-magnitude differences rather than subtle shifts. Moreover, the median spectral count fold change correlated well with true peptide load fold change, supporting the use of spectral counts as a semi-quantitative measure. Thus, the ePDF-based chi-square test is a valuable tool for label-free LC-MALDI proteomics.

### 3.2. Cell Fractionation Enriches Functionally Distinct Morphotypes

Centrifugation in Percoll density gradient effectively separated coelomocytes and CE cells into fractions with distinct morphological and functional properties. Coe1 (roundish cells, 58%) was unable to form cell nets in vitro, whereas Coe4 (enriched in large agranulocytes, 55%) readily formed nets and clots—a behavior reminiscent of the clot reaction described in echinoderm coelomocytes [19]. This functional dichotomy likely reflects different roles in immune defense and wound healing: the petaloid agranulocytes (Coe4) may represent an activated state or a specialized subset responsible for encapsulation and clot formation, while the roundish cells (Coe1) might be resting or non-phagocytic forms.

In CE fractions, the major cell types were ciliated cells (CE1, 46%; CE4, 52%) and small epithelial cells with a high nuclear-to-cytoplasmic ratio (SECs-2 in CE1, 30%; SECs-1 in CE-W, 50%). The tendency of CE4 ciliated cells to form aggregates, unlike those in CE1, suggests that fraction 4 contains a more adhesive subpopulation. Importantly, the CE-W subpopulation, which is mechanically detached during CE isolation, is highly enriched in SECs-1—the proposed coelomocyte precursors [7]. This enrichment makes CE-W a critical target for identifying early differentiation markers.

### 3.3. Proteomic Profiles Reflect Functional Specialization

Despite the morphological and functional differences between Coe1 and Coe4, their overall proteomes were surprisingly similar. Only a handful of proteins were significantly upregulated in Coe4, including alpha-actinin [20,21], plastin-2 [22,23], and dynein [24]. These are all involved in cytoskeletal dynamics–actin bundling and microtubule-based transport. The upregulation of these proteins in Coe4 aligns with the dramatic shape changes and motility required for cell net formation. Of interest is major vault protein (MVP), a component of large ribonucleoprotein particles found in Coe4 [25,26]. The lack of significant differences between the proteins of these fractions raises the question of whether these cell types represent the same lineage or are distinct. Comparison of fractions CE1 and CE4 also revealed minor differences in protein sets and morphological parameters, suggesting a common origin. These hypotheses will be tested in future studies.

Myosin heavy chain striated muscle-like (MHSM) [27,28] was upregulated in CE1. To date, among echinoderms, obliquely striated muscle fibers have only been found in ophiuroids and crinoids [29]. In contrast, microtubule–actin crosslinking factor 1 (MACF1), a large protein with numerous spectrin and leucine-rich repeat domains involved in the organization of the cytoskeleton and adhesion complexes [30], was upregulated in CE4. Additionally, several ribosomal proteins that also exhibit extraribosomal functions were upregulated in this fraction [31,32].

In contrast, the comparison between Coe4 and CE-W revealed extensive differences, consistent with their divergent cellular compositions: mature petaloid agranulocytes versus small, undifferentiated epithelia-derived cells. The CE-W fraction expressed higher levels of catenins (alpha (CA2) and beta CATB) and several adhesion molecules (e.g., neurofascin-like (NEU), a cell adhesion molecule involved in neuron–neuron adhesion, neurite fasciculation, and the outgrowth of neurites [33]; Sushi domain-containing protein (SP2), known to be a component of septate junctions (SJs) and cell–cell junctions, plays roles in paracellular permeability and barrier function in the epithelia of invertebrates, acting as a developmental protein [34,35,36]). The enrichment of catenins, which are central to adherens junctions and Wnt signaling, is clearly intriguing. In many animals, beta-catenin acts as a transcriptional co-activator in the Wnt pathway, regulating proliferation and differentiation [37,38]. Its upregulation in CE-W may indicate that these cells are poised to respond to Wnt signals, possibly from the underlying CE or from the coelomic fluid. Wnt signaling has been implicated in regeneration and cell fate decisions in echinoderms [39].

Notably, the CE-W set includes a broader spectrum of proteins than CE4; however, the differences are not as substantial as those observed in the previous case, suggesting a closer relationship between CE-W and CE.

### 3.4. Candidate Surface Markers: A Toolkit for Lineage Tracing

Among the 31 predicted membrane proteins that met our selection criteria, representatives from several superfamilies of transmembrane proteins stand out as promising markers for distinguishing coelomocytes, CE, and CE-W. Additionally, other proteins from these groups were identified in the proteome, occurring in all studied cell fractions (“common” in Table 1).

**Integrins**. Integrin heterodimers containing β1 together with α8 or α9 subunits are restricted to Coe cells, consistent with the mesenchymal-like, migratory phenotype of this population. The enrichment of α9β1 in coelomocytes supports a role in immune cell trafficking, as α9β1 is associated with activating signaling pathways that facilitate cell migration [40]. In contrast, α8β1 heterodimers may be involved in developmental processes, injury responses, and the mesenchymal–epithelial transition [41]. In our study, no alpha integrin subunits were detected in CE by proteomic study. This contrasts with results obtained in another study by PCR, a more sensitive technique [42], and is probably connected with the common cell types in CE and Coe. The beta1B subunit, a possible partner of the alpha integrin subunit, was present in CE in sufficient quantities. This raises the question of the beta1B integrin’s partner in CE.

**Three-finger proteins (TFPs) of the Ly6/uPAR family**. Four Ly6/uPAR paralogues—lystar-2, lystar-3, lystar-7 and lystar-8—are found among the surface-annotated proteins. Lystar-2 is expressed in CE-W cells, whereas lystar-3, lystar-7 and lystar-8 characterize the Coe population. GPI-anchored TFPs are involved in immunity and cell signaling [43,44]. The *A. rubens* genome comprises 48 genes containing LU domains [40]. One of the transcribed TFPs, Lystar5, may play a role in regulating neuromuscular transmission or coelomocyte homeostasis [45]. As GPI-linked three-finger proteins, these molecules are ideally positioned to modulate integrin activity within membrane microdomains [40]. Their cell-type-specific deployment suggests that different TFPs may modulate adhesion and migration in a context-dependent manner. Future validation should focus on Lystar2 as a tool for isolating SECs-1 cells from mixed populations.

**Scavenger receptor cysteine-rich (SRCR) domain proteins**. SRCRP-2 is detected on CE and CE-W cells, SRCRP-6 on CE-W cells, and SRCRP-4 on coelomocytes. The SRCR superfamily encompasses pattern-recognition receptors and soluble or membrane-bound proteins implicated in innate immunity and cell adhesion [46,47]. In Asterina pectinifera, SRCR proteins act as opsonins and involved in aggregation of coelomocytes [48]. The mutually exclusive expression of SRCRP4 (coelomocytes) and SRCRP6 (CE-W) suggests that they may label distinct functional stages: SRCRP4 on mature phagocytes, and SRCRP6 on precursor cells that have not yet acquired full opsonic capacity. Their differential distribution suggests that each cell type is equipped with a distinct set of immune or adhesion sensors.

**LDL receptor-related proteins**. LDLRPs showed a similar pattern: LDLRP12-1 was CE-W-specific, and a longer LDLRP (without CUB domains) was coelomocyte-specific. The presence of CUB domains in LDLRP12-1 is characteristic of proteins involved in complement regulation and developmental signaling [49]. Its restriction to CE-W reinforces the idea that this subpopulation contains cells with a distinct signaling or adhesive phenotype.

**Tetraspanins**. Tetraspanins (CD151A, TET7, TET9, TET15, TET18) were broadly expressed but with some subpopulation preferences. CD151A, a member of the CD63 subfamily, was found in both CE and CE-W but not in coelomocytes. Tetraspanins are known to organize membrane microdomains and regulate exosome biogenesis [50,51,52]. The presence of CD151A on CE and CE-W cells may relate to epithelial integrity or to the release of extracellular vesicles that modulate the coelomic environment [53].

**G-protein-coupled receptors**. The metabotropic glutamate receptor MGR4 (mGluR4) [54] is a surface marker of coelomocytes. Although classically studied in neurons, mGluR4 can modulate cAMP signaling and cytoskeletal dynamics in non-neuronal contexts, providing an additional layer of chemotactic control.

Of interest is the identification of a receptor tyrosine kinase (Tie-2, TPKRT2) [55], a receptor-type tyrosine phosphatase (RTPPF) [56], and plexin-A4 (PLEA4) in coelomocytes. Coelomocytes harbor a receptor tyrosine kinase (Tie-2, TPKRT2) [55], a receptor-type tyrosine phosphatase (RTPPF) [56] and plexin-A4 (PLEA4). The presence of counterbalancing kinase and phosphatase activities suggests that coelomocytes continuously integrate attractive and repulsive cues during migration. Plexin-A4 is a high-affinity receptor for class-1 semaphorins [57]. Its ligand semaphorin-1A (SEM1A) is also produced by coelomocytes, suggesting an autocrine or paracrine loop. Semaphorin-1A-plexin-A4 signaling typically triggers F-actin disassembly, growth cone collapse and repulsive guidance; in non-neuronal cells the same axis can limit or direct migration and modulate integrin activation [58]. The parallel presence of arachidonate 5-lipoxygenase (A5L) is notable because leukotrienes are potent chemoattractants that can amplify directional migration and adhesion [59]. The co-expression of semaphorin/plexin-repulsive machinery and a leukotriene-generating system in Coe cells indicates a highly regulated switch between stop and go signals, possibly allowing these cells to navigate complex tissue terrains.

### 3.5. Inferred Cellular Origins of Coelomic Fluid Proteins

Proteins previously identified in *A. rubens* coelomic fluid [60] exhibited differential abundance across the studied cell populations. Specifically, short-chain collagen protein C4-like and Lystar-3 appear associated with coelomocytes; Lystar-2 and scavenger receptor cysteine-rich domain superfamily protein-6 are linked to CE-W cells; multicopper oxidase is found in both coelomocytes and CE-W cells; hephaestin-like protein and low-density lipoprotein receptor-related protein are present in cells common to CE and CE-W; while Lystar-6, the putative defense protein astreelin, and scavenger receptor cysteine-rich domain superfamily protein-7 may be produced by all cell populations.

## 4. Materials and Methods

### 4.1. Animal Manipulation

Experiments were performed at the Biological Station of the Zoological Institute, Russian Academy of Sciences, on Cape Kartesh (Kandalaksha Bay, the White Sea), in September 2021–2023. Intact *A. rubens* L. (Asteroidea, Echinodermata) specimens, 10–12 cm in diameter, were collected off Fettakh Island and kept in cages at a depth of 3–5 m throughout the experimental period. They were fed ad libitum with a diet of mussels.

### 4.2. Isolation of Circulatory Coelomocytes

The coelomic fluid (CF) was collected after cutting off an arm tip and filtering the fluid through a nylon gauze into a test tube containing a saline solution free of Ca^2+^ and Mg^2+^ (CMFSS, [61]), supplemented with 15 mM EDTA (anticoagulant buffer) [5]. The cells were pelleted by centrifugation at 550× *g* for 10 min and washed twice in CMFSS. For each replicate, circulatory coelomocytes were pooled from four freshly caught starfishes with a diameter of 10–12 cm, yielding approximately 200 × 10^6^ cells.

### 4.3. Isolation of Coelomic Epithelium (CE) Cells (Epitheliocytes)

Fragments of the CE were detached with forceps from the inner surface of the aboral body wall of the arm, placed in CMFSS for 20 min, and then transferred to a 0.1% crab hepatopancreas collagenase solution (Biolot, St. Petersburg, Russia) in CMFSS for 15 min, with periodic pipetting to obtain the dissociated cells. The remaining washing CMFSS solution from this step contained a significant number of cells, classified as a distinct subpopulation of weakly attached cells (CE-W) [5,6]. These cells were collected and analyzed separately. The CE-W cell preparation and dissociated CE cells were filtered through nylon gauze, pelleted from the suspension by centrifugation at 550 *g* for 10 min, and washed twice with CMFSS. Approximately 500 × 10^6^ CE cells and 100 × 10^6^ CE-W cells were isolated from four freshly caught starfishes with a diameter of 10–12 cm. For each replicate, CE and CE-W cells were pooled from four freshly caught starfishes with a diameter of 10–12 cm, yielding approximately 500 × 10^6^ CE cells and 100 × 10^6^ CE-W cells.

### 4.4. Cell Separation in Discontinuous (Step) Percoll Density Gradients

CF and CE cell separation was performed using discontinuous Percoll density gradients (steps 50%–45%–40%–35%–30%–25%, 1 mL each) according to the manufactory protocol (GE Healthcare, Uppsala, Sweden) with some modification. To prepare a stock isotonic Percoll (SIP) solution, 9 parts (*v*/*v*) of Percoll were mixed with 1 part (*v*/*v*) of 10× CMFSS solution. The SIP was then diluted to create lower density gradients by adding CMFSS, which were layered into 15 mL polycarbonate centrifuge tubes (Sarstedt, Germany), starting with the densest at the bottom. A coelomocyte suspension was layered on top in 0.5 mL of CMFSS/5 mM EDTA (approximately 35 × 10^6^ cells per gradient), and the tubes were centrifuged at 400× *g* for 20 min at 8 °C using a swing-out bucket. CE cell suspensions were layered in 0.5 mL of CMFSS (approximately 75 × 10^6^ CE cells per gradient), and centrifuged at 400× *g* for 25 min at 8 °C using a swing-out bucket.

The visible layers of cells at phase boundaries were collected with a Pasteur pipette, transferred into the tubes containing 7 mL CMFSS, and then centrifuged at 550× *g* for 10 min at 8 °C. The cells were then resuspended in 1 mL of CMFSS. The number of cells in each fraction was counted using a hemocytometer, and the cell suspensions were subdivided for fixation, functional tests, and proteomic analysis. The total number of cells in all fractions was summed up, and the proportion of cells in each fraction was calculated as a percentage.

### 4.5. Fluorescent Staining of CF and CE Cell Suspensions

The circulatory cells from CF, as well as CE and CE-W cells, were fixed with 4% paraformaldehyde (PFA), and placed onto coverslips coated with poly-L-lysine (Sigma-Aldrich, St. Louis, MO, USA) (1 h), and then stained with DAPI. The efficiency of cell fractionation in Percoll density gradients was assessed by comparing the proportion of distinct cell morphotypes. Preparations were examined in transmitted and fluorescence light (DAPI staining) using a ×100 objective lens magnification under an Axiovert 200M microscope (Carl Zeiss, Jena, Germany) equipped with a Leica DFC420 digital camera. We employed the previously established terminology to characterize cell morphotypes [13,14,22], utilizing criteria such as cell and nucleus size and shape, presence or absence of granules, and the pattern and intensity of staining. Cell sizes were measured from images obtained with a camera with a known resolution. Cell counts in three parallel samples were conducted across several randomly selected microscopic fields, totaling no fewer than 400 cells analyzed in each sample.

### 4.6. Functional Test of Cell Fractions In Vitro

The details of the experiment regarding the characteristics of coelomocytes and CE behavior in vitro were previously described [14]. For live cell imaging in the present work, cells of individual fractions 1 and 4 of coelomocytes and CE were washed in CMFSS, precipitated by centrifugation at 550× *g* for 10 min, resuspended in sterile seawater, and plated onto 96-well plates at a volume of 100 μL of cells (0.5 × 10^6^ of coelomocytes and 0.8 × 10^6^ of CE cells per well). Images were acquired in transmitted light using an inverted Biolam microscope (LOMO. St. Petersburg, Russia), equipped with a camera, over a period of 4 h. Since CE cells did not exhibit any changes during an 18 h incubation in seawater—aside from the initial formation of cell aggregates in fraction 4 [14]—live cell imaging for the CE is not presented here.

### 4.7. Isolation of Membrane Proteins

A Thermo Scientific Mem-PER Plus Membrane Protein Extraction Kit (USA) was used to isolate membrane and membrane-associated proteins with modifications. Membrane proteins were extracted from fractions 1 and 4 of coelomocytes, fractions 1 and 4 of CE, and the CE-W suspension, enriched in SECs-1. Cell suspensions (approximately 20 × 10^6^ cells per fraction) were collected after cell resuspending in 1 mL of CMFSS, and sedimented in microcentrifuge for 7 min at 500× *g*. The pellets were resuspended in 0.5 mL of Permeabilization Buffer supplemented with protease cocktail and PMSF (up to 2 mM), vortexed briefly, and incubated for 10 min at 4 °C with constant mixing. The cells were than centrifugated for 15 min at 13,000× *g*. Cytoplasmic proteins were carefully removed and 0.3 mL of solubilization buffer was added to the pellets. The pellets were resuspended by pipetting up and down, incubated at 4 °C for 30 min with constant mixing and centrifuged at 13,000× *g* for 15 min. The supernatants, containing solubilized membrane and membrane-associated proteins, were aliquoted into new tubes for future research and frozen in liquid nitrogen. Membrane proteins isolated with kits included integral proteins from the plasma membrane, as well as proteins from the endoplasmic reticulum, Golgi apparatus, lysosomes, and secretory vesicles. They also encompassed proteins from the outer and inner membranes of mitochondria and those associated with the nuclear envelope.

### 4.8. Protein Digestion, Mass Spectrometry and Database Search

Proteins were solubilized in 8 M urea. Proteins/SEPs were reduced with 10 mM DTT for 30 min, alkylated with 25 mM IAA for 45 min in the dark at room temperature, and then digested with modified trypsin (Trypsin Gold, Promega, Madison, WI, USA) overnight at 37 °C with a trypsin-to-protein ratio of 1:20 (*w*/*w*) in less than 1 M urea. All digests were desalted and concentrated with Strata-X 30 mg solid-phase extraction tubes (Phenomenex, Torrance, CA, USA). Eluted digests were supplemented with 10 μL of 50 mg/mL D-glucose to aid subsequent rehydration and dried in a rotor vacuum evaporator (Martin Christ, Osterode am Harz, Germany). Then, digests were resuspended in 25 μL of 1% (*v*/*v*) formic acid in water and filtered through 0.2 μm PVDF filter. Peptides were separated with a Chromolith CapRod RP-18e HR reversed-phase column (0.1 mm × 150 mm, Merck, Darmstadt, Germany) on a nano LC system (Eksigent NanoLC Ultra 2D+ system, Sciex, Darmstadt, Germany). A total peptide amount of 1000 ng was loaded and separated using a linear gradient of 6.5–43% B over 44 min at a flow rate of 400 nL·min^−1^. The mobile phases used were A, water with 0.2% (*v*/*v*) TFA and B, 80% (*v*/*v*) acetonitrile in water. The column was operated at a room temperature of 22–24 °C. The effluent from the column was mixed with matrix solution (CHCA 9 mg·mL^−1^, 0.2% (*v*/*v*) TFA in 85% acetonitrile) containing two calibration standards: bradykinin 2–9 (30 pM·mL^−1^) and ACTH 18–39 (60 pM·mL^−1^), at a flow rate of 2.4 μL·min^−1^. A micro-fraction collector was used to deposit 1 mm spots every 2 s, and a total of 704 spots were collected in a 44 × 16 array for each nano LC run. The column was washed with a gradient (0–100–100% B for 5 min and 2 min respectively, at a flow rate of 600 nL·min^−1^) and equilibrated to 0% B for 3.5 min before subsequent injections.

The fractionated samples were analyzed with a TOF/TOF 5800 System (Sciex) instrument operated in the positive ion mode. The MALDI stage was set to continuous motion mode. MS data was acquired at 2800 laser intensity with 500 laser shots/spectrum (250 laser shots/sub-spectrum) and MS/MS data were acquired at 3700 laser intensity with a DynamicExit algorithm and a high spectral quality threshold or a maximum of 1000 laser shots/spectrum (250 laser shots/sub-spectrum). Up to 30 top precursors with S/N > 40 in the mass range 750–3500 Da were selected from each spot for MS/MS analysis.

The Protein Pilot 5.0.1 software (Sciex, Darmstadt, Germany) with the Paragon algorithm in thorough mode was used for the MS/MS spectra search against the *A. rubens* genome-derived protein database GCF_902459465.1 downloaded from the NCBI. Carbamidomethyl cysteine was set as a fixed modification. The database also incorporated a list of common contaminants and decoy sequences generated with DecoyPYrat [62]. Protein grouping, alignment of multiple runs and false discovery rate (FDR) analysis was conducted with custom Python v.3.8 script. Proteins passing the 1% global FDR threshold were accepted. The proteomics dataset and search database are available on Zenodo: https://doi.org/10.5281/zenodo.17174375.

### 4.9. Label-Free Quantitation of Protein Abundances

Proteins were included in the final list of accepted identifications only if they were detected in all replicates of at least one experimental group (Table S3). All samples were analyzed in triplicate, with each replicate representing a sample pooled from four animals. Differential abundance was assessed using a chi-square test of symmetry to compare the observed spectral count frequencies between experimental and control group to a hypothesized 1:1 ratio. The chi-square statistic used is

(1) $$\chi_{c}^{2}=\frac{{\left({\bar{sc}}_{e}-{\bar{sc}}_{c}\right)}^{2}}{{\bar{sc}}_{e}+{\bar{sc}}_{c}},$$

where sce and scc are the mean spectral counts over replicates in the experimental and control groups, respectively. Spectral counts are defined as the sum of peptide-spectrum matches (PSMs) for a given protein. Assuming that the minimum observed PSM count for a protein identified by a single peptide was 6, any missing values were imputed with a value of 5 PSMs. This imputation reflects a conservative estimate that the protein was present but below the limit of detection, preventing artificial inflation of fold changes. A protein was classified as differentially abundant if its spectral count ratio in the experimental and control groups deviated significantly from 1:1 (*p* < 0.001). Instead of relying on the theoretical chi-squared distribution (with 1 degree of freedom), *p*-values were calculated using the empirical probability density function (ePDF) and adjusted for multiple comparisons using the Benjamini–Hochberg procedure. This ePDF was derived from the distribution of 11,910 chi-square statistical values obtained from 12 LC-MALDI runs of identical peptide loads (1 µg Pierce HeLa protein digest standard). To generate the empirical null distribution, the 12 runs were permutated into 18 groups of three (mimicking triplicate experimental and control groups, Table S4). For each protein in each permutated group, the mean spectral counts were calculated, and the chi-square statistical test was computed based on Equation (1). This averaging step reduced inter-run variability and improved precision. Averaging was justified because the distribution of spectral counts across replicates is normal (Figure 2c). Then, the method was validated by triplicate LC-MALDI runs of four proteomic mixtures of HeLa and Gram-negative bacterium *Serratia proteamaculans* (SePr) digests with total protein amounts of 600 ng. The set of mixtures was: SePr 500 ng and HeLa 100 ng, SePr 400 ng and HeLa 200 ng, SePr 200 ng and HeLa 400 ng, SePr 100 ng and HeLa 500 ng. This set allowed assessment of 1.25-, 2-, 2.5-, 4-, and 5-fold changes in protein abundance. Proteomics datasets are available on Zenodo: https://doi.org/10.5281/zenodo.17243179 and https://doi.org/10.5281/zenodo.20042848.

### 4.10. Bioinformatic Analysis

STRING v.11.5 [63] was used for functional annotation of *A. rubens* proteome and enrichment analysis, Cytoscape v.3.10.0 [64] for visualization of protein interaction clusters, and DeepLoc v.2.01 [65] for prediction of subcellular location.

### 4.11. Statistics

The proportion of CF and CE cells morphotypes in different fractions of Percoll density gradient are shown in Figure 1 as a pie chart; full statistics are presented in the Table S1 File. The data expressed as mean ± SEM (*p* < 0.05) (*n* = 3); all data were processed statistically by ANOVA with Tukey’s HSD multiple comparison test to determine significant differences (*p* < 0.05), using the STATISTICA 7.0 Software.

## 5. Conclusions

This study provides the first proteome-scale survey of surface proteins enriched in distinct cell fractions from *A. rubens* coelomocytes and coelomic epithelium. By combining density-gradient fractionation with proteomic profiling, we generated a list of 31 candidate markers, including integrins, three-finger proteins, SRCR proteins, and LDLR-related proteins. Among these, Lystar2, SRCRP6, and LDLRP12-1 are particularly noteworthy due to their unique or high enrichment in the CE-W fraction, which contains the presumptive coelomocyte precursors (SECs-1). As this study utilizes proteomics and bioinformatics data, the identified differentially expressed proteins are considered only candidate markers for individual cell types. The most promising candidates will be further confirmed as marker proteins using PCR, FISH, and immunofluorescence analysis. Nevertheless, these markers open the door to lineage tracing studies that can finally resolve the long-standing question of whether coelomocytes arise from dedicated stem cells, from dedifferentiation of epithelial cells, or from a combination of both. More broadly, our approach serves as a template for cell surface marker discovery in non-model invertebrates where genomic resources are limited but biological questions are rich.

## Figures and Tables

**Table 1:** Representatives of superfamilies among differentially abundant transmembrane proteins.

	Coe1/Coe4	CE-W	Coe/CEW	Common	CE/CE-W
**Integrins**	IA9-1		IA9-2 * IA8 * IB1 *	IB1B *	
**TFPs**		Lys2	Lys7 Lys8	LYS3 Coe * LYS9	
**SRCRP**	SRCRP4 *	SRCRP6	SRCRP		
**LDLRP**	LDLRP *	LDLRP12.1			LDLRP12.2 LDLRP.2
**Tetraspanins**				TET18 TET9 TET15	TSPAN7 CD151A
**G-protein-coupled receptors**	GNBPA13 MGR4	GNBPGQA			

LDLRP—Low-density lipoprotein receptor gene family. SPCRP—Scavenger receptor cysteine-rich domain superfamily proteins. TFPs—Lu domain-containing three-finger proteins. *—Major proteins.

## Data Availability

The mass spectrometry proteomics data have been deposited on Zenodo: https://doi.org/10.5281/zenodo.17243179 and https://doi.org/10.5281/zenodo.20042848. The functional assay has been deposited in Mendeley: https://doi.org/10.17632/7yx5rrr854.1.

## Supplementary Materials

The following supporting information can be downloaded at https://www.mdpi.com/article/10.3390/ijms27146292/s1.
