# Supplementary material for: Proteomic Profiling of Density-Gradient Fractions Reveals Candidate Cell Surface Markers in Subpopulations of Starfish Asterias rubens Coelomocytes and Coelomic Epithelial Cells
Source: Int J Mol Sci. 2026 Jul 15;27(14):6292. doi: 10.3390/ijms27146292 (PMC13409769; doi:10.3390/ijms27146292)
Supplement: Supplementary file 1 [file ijms-27-06292-s001.zip › Table S1.pdf]

Table S1. The proportion (%) of distinct cell morphotypes in selected Percoll fractions

	Cell type	Fraction 1	Fraction 4
**Coelomocytes**	Small coelomocytes with discreetly and densely stained nuclei (SCoe-1+2)	2, 5 ± 0,5	1.6 ± 1,5
	Roundish cells (R)	57.8 ± 2,1	5,3 ± 3,2
	Small agranulocytes (SAgr)	11,29 ±1,1	19,22 ± 1,3
	Large agranulocytes (Agr)	16 ± 1,6	54,8 ± 4,9
	Large granulocytes (Gr)	10,9 ± 1,2	16,7 ± 0,9
	2- or 3-nuclear cells (2-N)	1,4 ± 0,5	2,9 ± 0,4
	**Cell type**	**Fraction 1**	**Fraction 4**
**Coelomic epithelium**	Small CE cells with discreetly stained nuclei (SCEs-1)	1,7 ± 0,5	0,2 ± 0,1
	Small CE cells with densely stained nuclei (SCEs-2)	30,6 ± 2,1	7,6 ± 0,9
	Ciliated cells	45,9 ± 1,5	52,4 ± 2,2
	Small agranulocytes (CEAgr)	14,5 ± 1,7	25 ± 1,5
	Large agranulocytes (Agr)	3,8 ± 0,7	2,5 ± 0,3
	Large granulocytes (Gr)	3,7 ± 0,6	12,3 ± 1,7
	**Cell type**	**Control**	
**CE-W**	Small CE cells with discreetly stained nuclei (SCEs-1)	49,9 ± 2,3	
	Small CE cells with densely stained nuclei (SCEs-2)	6,3 ± 1,9	
	Ciliated cells	19 ± 1,7	
	Small agranulocytes (CEAgr)	14,4 ± 1,5	
	Large agranulocytes (Agr)	8,7 ± 0,7	
	Large granulocytes (Gr)	1,6 ± 0,6	
