# Supplementary material for: Proteomic Profiling of Density-Gradient Fractions Reveals Candidate Cell Surface Markers in Subpopulations of Starfish Asterias rubens Coelomocytes and Coelomic Epithelial Cells
Source: Int J Mol Sci. 2026 Jul 15;27(14):6292. doi: 10.3390/ijms27146292 (PMC13409769; doi:10.3390/ijms27146292)
Supplement: Supplementary file 1 [file ijms-27-06292-s001.zip › Table_S2.pdf]

Table S2. Morphometry of cells identified in coelomocytes (Coe), coelomic epithelium (CE) fractions and in weakly attached CE cells (CE-W)

	**Cell type**	**Diameter of nuclei (μm)**	**Diameter of cell (μm)**	**Cell characterictic**
	**Coelomocyte-specific**			
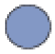	Small coelomocytes with densely stained nuclei (SCC-2)	3.85±0.32	4.2±0.2	Cells with a high nuclei-cytoplasmic ratio
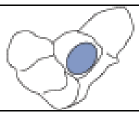	Small agranulocytes (SAgr)	3.75±0.46	7,33±0.2	Petaloid cells
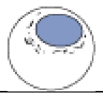	Roundish cells (R)	4,3 ± 0,1	8.2 ±0.2	round cells with granulas (grains) in the near-nuclear region
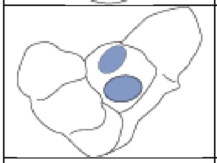	2- or 3-nuclear cells (2-N)	4,25±0,75	12,4±0,5	Petaloid cells
	**CE/CE-W-specific**			
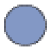	Small CE cells with densely stained nuclei (SCEs-2)	2.8±0.2	3.3±0.2	Cells with a high nuclei-cytoplasmic ratio and invisible cytoplasm with densely stained nuclei
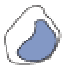 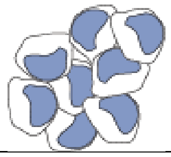	Flagelated cells type 1 (FC-1)	3±0.5	4,8±0,17	Small agranulocytes with irregularly shaped nuclei; in Fr 1 – they are single cells; In Fr 4 – they are tend to form the aggregates (FC-4s)
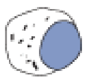	Small agranulocytes (CEAgr)	3.2±0.6	6±0,33	Cellswith roundish nuclei; In Fr 4 – they are tend to form the aggregates
	**Common types**			
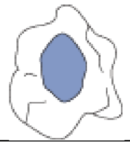	Large agranulocytes (Agr)	4,18±0,7	In CE and CE-W: 9,5±0,33 In CF: 11,7±0,7	

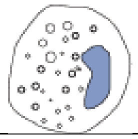	Large granulocytes (Gr)	4.21±0.9	In CE and CE-W: 9,3±0,5<br>In CF: 10,84±0,28 µm	Eosinophilic cells with granules
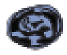	Small cells (SCs)	Roundish: 4,06±0,2<br>Oval: 4×6,06±0,5	Roundish: 4,06±0,2<br>Oval: 4×6,06±0,5	small cells with a high nuclear-cytoplasmic ratio, discretely stained nuclei and invisible cytoplas
